# Inflammatory projections after focal brain injury trigger neuronal network disruption: An ^18^F-DPA714 PET study in mice

**DOI:** 10.1016/j.nicl.2018.09.031

**Published:** 2018-10-01

**Authors:** Sanae Hosomi, Tadashi Watabe, Yuki Mori, Yoshihisa Koyama, Soichiro Adachi, Namiko Hoshi, Mitsuo Ohnishi, Hiroshi Ogura, Yoshichika Yoshioka, Jun Hatazawa, Toshihide Yamashita, Takeshi Shimazu

**Affiliations:** a Department of Traumatology and Acute Critical Medicine, Osaka University Graduate School of Medicine, 2-15 Yamada-oka, Suita-shi, Osaka 565-0871, Japan; b Department of Nuclear Medicine and Tracer Kinetics, Osaka University Graduate School of Medicine, 2-15 Yamada-oka, Suita-shi, Osaka 565-0871, Japan; c Medical Imaging Centre for Translational Research, Osaka University Graduate School of Medicine, 2-15 Yamada-oka, Suita-shi, Osaka 565-0871, Japan; d Centre for Information and Neural Networks (CiNet), National Institute of Information and Communications Technology (NICT) and Osaka University, 1-4 Yamada-oka, Suita-shi, Osaka 565-0871, Japan; e Department of Molecular Neuroscience, Osaka University Graduate School of Medicine, 2-15 Yamada-oka, Suita-shi, Osaka 565-0871, Japan; f Division of Gastroenterology, Department of Internal Medicine, Kobe University Graduate School of Medicine, 5-2 Kusunoki-cho 7, Chuo-ku, Kobe-shi, Hyougo 650-0017, Japan

**Keywords:** Translocator protein-positron emission tomography, Traumatic brain injury, Neurodegeneration, Neuroinflammation

## Abstract

Due to the heterogeneous pathology of traumatic brain injury (TBI), the exact mechanism of how initial brain damage leads to chronic inflammation and its effects on the whole brain remain unclear. Here, we report on long-term neuroinflammation, remote from the initial injury site, even after subsiding of the original inflammatory response, in a focal TBI mouse model. The use of translocator protein-positron emission tomography in conjunction with specialised magnetic resonance imaging modalities enabled us to visualize “previously undetected areas” of spreading inflammation after focal cortical injury. These clinically available modalities further revealed the pathophysiology of thalamic neuronal degeneration occurring as resident microglia sense damage to corticothalamic neuronal tracts and become activated. The resulting microglial activation plays a major role in prolonged inflammatory processes, which are deleterious to the thalamic network. In light of the association of this mechanism with neuronal tracts, we propose it can be termed “brain injury related inflammatory projection”. Our findings on multiple spatial and temporal scales provide insight into the chronic inflammation present in neurodegenerative diseases after TBI.

## Highlights

• TSPO-PET tomography enables the assessment of longitudinal neuronal inflammation
• Inflammatory responses at the cortical injury site diminish after about 1 week
• The ipsilateral thalamus exhibits remote neuroinflammation for up to 14 weeks
• Microglial activation is associated with remote chronic degeneration
• Inflammation expands to remote sites via damaged cortico-thalamic projections

## Graphical abstract

TSPO-PET detects two types of distinct inflammation occur after focal cortical injury: 1) cortical lesions respecting stable lesion boundaries, complete scars and rapid neuronal loss of injured tracts, and 2) thalamic lesions exhibiting delayed inflammatory response and long-term neuronal degeneration of uninjured tracts

**Figure:**
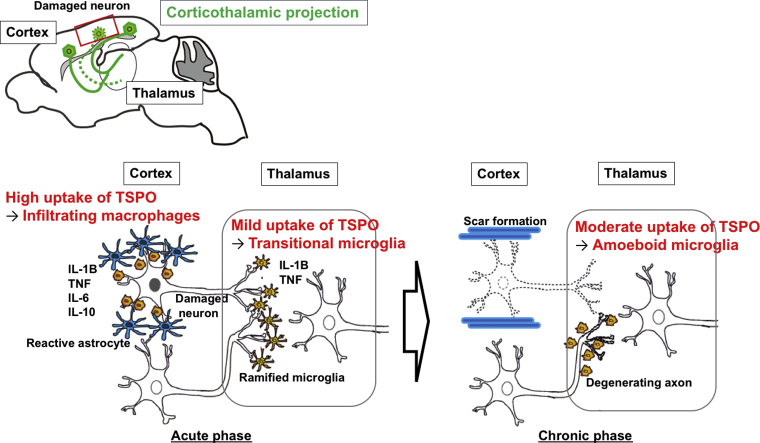

## 1 Introduction

Traumatic brain injury (TBI) is an increasingly prominent public health and societal issue worldwide (Majdan et al., 2016). It is also associated with increased mobility, mortality (Brooks et al., 2013), and decreased life expectancy (Brooks et al., 2015). Individuals with TBI too often incur substantial health care costs, including caregiver-related costs, as well as loss of productivity (Farhad et al., 2013; Ma et al., 2014; Olesen et al., 2012). TBI is one of the leading causes of disability in the field of neuropathology. Previous history of acute mild TBI is the strongest risk factor for neurodegenerative conditions among contact sport athletes and war veterans (Nordström et al., 2014; Vincent et al., 2014) and is a suspected factor in various chronic neurodegenerative conditions, including traumatic encephalopathy, Alzheimer's, and Parkinson's disease (Chauhan, 2014).

Typically, inflammation progresses from an acute to a chronic phase and then resolves (Chen and Nuñez, 2010). Unresolved inflammation, however, significantly contributes to the pathogenesis of various diseases (Nathan and Ding, 2010), including neurodegenerative and psychiatric maladies. Traditional histological and immunohistochemical approaches have identified limited aspects of acute and chronic inflammation in the brain post-TBI (Johnson et al., 2013). Some experimental and clinical evidence suggests that TBI can initiate chronic biochemical processes that lead to prolonged neuroinflammation or microglial activation, thus contributing to chronic neuropathy (Johnson et al., 2013; Loane et al., 2014). However, due to TBI variability and/or patient heterogeneity, the exact mechanism underlying brain damage-related chronic inflammation and its effects on neurodegeneration remain unclear (Russo and McGavern, 2016).

The inflammatory reaction in the brain involves a dramatic increase in the expression of translocator protein (TSPO), and this upregulation is considered a hallmark of neuroinflammation. Positron emission tomography (PET) is a molecular imaging technique that can quantify molecular targets in vivo (O'Brien et al., 2014), while proton magnetic resonance spectroscopy (^1^H-MRS) and tractography have the advantage of directly assessing the neurochemical profile and neural tracts, respectively, in specifically chosen regions (Saito et al., 2012; Fujiyoshi et al., 2013). The goal of the present study was to achieve a better understanding of the pathophysiology of chronic neuroinflammation, by studying a mouse model of TBI, by which the observation of anatomical changes can be coupled with precise control both of external mechanical force and environmental factors. Here, we hypothesized that resident microglia participate in neurodegeneration post-TBI and performed analyses using various clinically available modalities. As a result, we found that resident microglia in the ipsilateral thalamus sense damage to corticothalamic neuronal tracts and become activated, resulting in a delayed inflammatory process. Taking advantage of the high sensitivity of PET in conjunction with high-resolution MRI, we were able to identify specific pathologies relevant to non-resolving inflammation, associated with neurodegenerative disease.

## 2 Materials and methods

### 2.1 Animal preparation

Adult male C57BL/6 J mice (8–10-weeks-old) were purchased from Japan SLC Inc. (Shizouka prefecture, Japan) and housed in groups of three, on a 12-h light-dark cycle in standard cages, with food and water available ad libitum. Following anaesthesia with 0.3 mg/kg medetomidine hydrochloride (Domitol; Meiji Seika Pharma Co., Ltd., Tokyo, Japan), 4 mg/kg midazolam (Dormicum; Astellas Pharma Inc., Tokyo, Japan), and 5 mg/kg butorphanol (Vetorphale; Meiji Seika Pharma Co., Ltd.), mice were stabilised in a stereotaxic frame (NARISHIGE Group, Tokyo, Japan). Controlled cortical impact (CCI) was performed, as previously described (Ueno et al., 2012). The scalp was retracted, and a circular 4-mm craniotomy was performed using a drill, on the left side, with the centre at 0 mm anteroposterior and 2 mm lateral to the bregma. Cortical TBI was induced using a pneumatic impact device (Amscien Instruments, Richmond, VA, USA). Briefly, the dural surface was exposed and the tip of the impactor (diameter = 3 mm) was positioned over the centre of the opened surface. The tip was then lifted and lowered by 1 mm, and impact was induced at a speed of 4.0–4.5 m/s for 90 ms. Thereafter, the skull fragment was replaced, the scalp was sutured, and mice were allowed to recover. Mice that underwent sham operations (i.e., craniotomy without cortical traumatic injury) were used as controls. All procedures were in line with the ARRIVE guidelines and performed in accordance with the guidelines of Osaka University Graduate School of Medicine for the Care and Use of Laboratory Animals and approved by the institutional ethics committee.

### 2.2 Synthesis of ^18^F-DPA-714

^18^F-DPA-714 preparation was described in our previous publication (Kashiyama et al., 2016). Accordingly, ^18^F-fluoride in ^18^O-H_2_O was transferred to a UG-M1 synthesiser (Universal Giken, Kanagawa, Japan) and passed through an Accell Light QMA cartridge. We eluted the trapped ^18^F-fluoride from the Sep-Pak cartridge using a solution containing 33 mM K_2_CO_3_ (in 200 μL of water), acetonitrile (700 μL), and 23.3 mg of Kryptofix-222. We then transferred it to a reaction vessel and allowed for the complete evaporation of the liquid. Tosylate precursor (10 mg) in acetonitrile (900 μL) was added to the reaction vessel (including ^18^F-KF with Kryotofix-222), which was heated at 85 °C for 5 min to fluorinate ^18^F. We allowed the mix to cool down and subjected the crude mixture to semi-preparative reverse-phase high performance lipid chromatography (HPLC), using Xterra Prep MS C18 10-μm (7.8 × 300 mm) columns, in a mobile phase of 0.1 M aqueous ammonium acetate (NH4OAc) and CH3CN (40:60, *v*/v), at a flow rate of 4.0 mL/min. We collected the radioactive fraction corresponding to ^18^F-DPA-714 and vacuum evaporated it using a rotary evaporator. We resuspended the residue in sterile water (5 mL) and filtered it through a 0.22-μm Millipore Millex GV sterile filter into a sterile pyrogen-free evacuated vial. The radioactive end product was 3–5 GBq, with a specific activity of 800–1500 GBq/μmol and radiochemical purity >97%.

### 2.3 PET/computed tomography (CT) acquisition

A bolus of ^18^F-DPA-714 (10.1 ± 2.7 MBq/0.1 mL) was injected into the cervical vein under 2% isoflurane anaesthesia. All acquisitions (10 min static scans acquired 20 min post-injection) were performed with an Inveon small animal PET/CT scanner (Siemens Healthineers, Erlangen, Germany) (Wang et al., 2014a, Wang et al., 2014b). The protocol for static PET imaging was determined based on a previous study, as the whole-body distribution became stabilised at this time-point (Boutin et al., 2007; Chauveau et al., 2009; Vicidomini et al., 2015). Longitudinal ^18^F-DPA-714 PET studies were performed on TBI mice (body weight = 23.9 ± 3.5 g) at day 1, 4, 7, 14, 21, 28, 42, 63, and 98 post-injury (*n* = 3–4 at each point, total *n* = 31), and on sham-operated control mice at day 1, 4, 7, 14, 21, 28, 42, 63, and 98 post-injury (n = 3–4 at each point, total *n* = 30). Different animals were used for each time-point. PET images were reconstructed with three-dimensional (3-D) ordered-subset expectation maximization (OSEM3D), followed by maximum a posteriori (MAP) estimation (16 subsets, 2 OSEM3D iterations, and 18 MAP iterations) with attenuation and scatter correction. The image matrix was 128 × 128 × 159 voxels, with a voxel size of 0.776 × 0.776 × 0.796 mm^3^. Using fused PET/CT and MR images for guidance, we manually defined 3-D ellipsoidal regions of interest on the cortical lesion site, ipsilateral thalamus, and cerebellum, using the PMOD software (version 3.404; PMOD Technologies, Zurich, Switzerland; see Inline Supplemental Fig. 1). The maximum and mean standardized uptake values (SUVmax and SUVmean, respectively) were calculated by dividing the tissue radioactivity concentration (Bq/mL) by the injected radioactive dose (Bq/g). Uptakes were compared between TBI and sham groups using the target-to-cerebellum ratio (ratio of SUVmax of the target to SUVmean of the cerebellum) and SUVmax.

We also performed macrophage depletion studies to evaluate the influence for TSPO uptakes on PET. A 100-μL bolus of clodronate liposomes (6.5 μg/μL; Katayama Chemical, Osaka, Japan) or vehicle was randomly injected into a total of 6 CCI mice via the tail vein, 1 h post-CCI, by modifying a previously reported method (Mori et al., 2014). ^18^F-DPA-714 PET was then performed 1 week post-CCI. SUVs were measured at two locations, based on the in vivo MR imaging results, namely, at 1.1 mm (cortex) and 2.9 mm (optic thalamus) from the brain surface.

### 2.4 MRI and magnetic resonance spectroscopy

MR spectroscopy (MRS) was conducted for analysing the thalami during the chronic phase, using an 11.7-T scanner (AVANCE II 500WB; Bruker Biospin, Ettlingen, Germany) and a 15-mm inner-diameter coil under isoflurane anaesthesia, as previously described (Saito et al., 2012). In vivo ^1^H-MRS was performed to measure the detailed neurochemical profile of TBI-and sham-operated animals (*n* = 3 per group) at 4, 6, 9, and 14 weeks after brain injury using the same animals at each time-point. Spectra were obtained from cubic voxels (2 × 2 × 2 mm^3^), encompassing both lateral thalami, using point-resolved spectroscopy with the following parameters: repetition time/echo time (TR/TE) = 3000 ms/10 ms, number of signals averaged (NSA) = 256, spectral width = 6 kHz, data size = 8 K, and acquisition time = 13 min. MR tractography was performed ex vivo on fixed brain tissue of TBI mice (*n* = 2) 1 week post-injury. Diffusion tensor imaging (DTI) was performed using a spin echo (SE) sequence with the following parameters: TR/TE = 5000 ms/16 ms, NSA = 4, field of view (FOV) = 15 × 15 mm, matrix size = 128 × 128, slice thickness = 0.5 mm, motion probing gradient = 6 directions, b-value = 1000 s/mm^2^, and acquisition time = 3 h 58 min. Fibre tracking was performed using dTV.II and VOLUME ONE software (Hiroshima City University, Hiroshima, Japan), and tract bundles were selected by determining the seed region.

### 2.5 Immunohistochemistry

Immunohistochemical analysis was performed according to standard protocols (Ueno et al., 2012). Briefly, mice (*n* = 3 per timepoint) were transcardially perfused with 4% PFA in 0.1 M phosphate buffer (PB) and their brains were dissected, post-fixed overnight at 4 °C, and subsequently immersed in 30% sucrose in phosphate buffered saline (PBS). Tissues were embedded in optimal cutting temperature compound, frozen on dry ice, and stored at −80 °C. Serial 20-μm-thick sections were cut on a cryostat and mounted on silane-coated adhesive slides (Matsunami Glass, Osaka, Japan). Sections were blocked with 5% bovine serum albumin and 0.1% Triton X-100 in PBS for 1 h, and subsequently incubated with the following primary antibodies: mouse anti-glial fibrillary acidic protein (anti-GFAP; 1:500; G-A-5; Sigma-Aldrich, St Louis, MO, USA), rabbit anti-TSPO (1:100; ab109497; Abcam, Cambridge, MA, USA), rabbit anti-ionized calcium binding adaptor molecule 1 (Iba1) (1:500; 019–19,741; Wako Chemicals, Richmond, VA) and rat anti-CD11b (1:100; MCA711; Bio-Rad, Hercules, CA, USA) overnight at 4 °C (Wang et al., 2014a, Wang et al., 2014b). Sections were washed thrice with 0.05% Tween 20 in PBS and incubated with the Alexa 488-conjugated and Alexa Fluor 568-conjugated goat anti-rabbit, −mouse, and -rat immunoglobulin G (1:500, Thermo-Fisher Scientific, Waltham, MA, USA) secondary antibodies for 1 h at 20–27 °C. Sections were counterstained with 4′,6-diamidino-2-phenylindole (DAPI; 1 μg/mL; Santa Cruz Biotechnology, Dallas, TX, USA). All images were acquired with a fluorescence microscope (BX51 or DP71; Olympus, Tokyo, Japan) or confocal laser-scanning microscope (FluoView FV1000; Olympus).

### 2.6 Giemsa staining

CCI-operated mouse brains (*n* = 3 per timepoint) were fixed by perfusion, trimmed, dehydrated in an ascending ethanol series, and embedded in paraffin. Samples were sliced into 5-μm-thick sections, deparaffinised, and rehydrated in a descending ethanol series. The sections were stained first with a May-Grünwald stain solution (Muto Pure Chemicals, Tokyo, Japan) in methanol for 30 min at approximately 25 °C and then with a Giemsa stain solution (Merck KGaA, Darmstadt, Germany) in 0.067 M PBS for 4 h at approximately 25 °C. After differential staining with 1% acetic acid and dehydration with isopropanol, the samples were dried and mounted on slides using Entellan (Merck KGaA) with xylene (Zaffaroni et al., 1999).

### 2.7 Electron microscopy

For histological evaluation of MRS results, CCI- and sham-operated mouse brains (*n* = 3 per group) at the 4-week injury timepoint were transcardially perfused with 2% PFA and 2.5% glutaraldehyde in 0.1 M PB (pH 7.4) and post-fixed at 4 °C overnight. The thalamus was isolated by trimming and cutting into 50-μm-thick sections, using a vibrating blade microtome (VT1000s; Leica, Wetzlar, Germany). Coronal sections showed the largest area of damage; therefore, contralateral coronal sections at the same level were selected, washed in 0.1 M PB, and arranged for electron microscopy (Koyama et al., 2013). All electron micrographs were observed and obtained using a transmission electron microscope (H-7650; Hitachi, Tokyo, Japan).

### 2.8 RNA isolation and quantitative real-time polymerase chain reaction (qRT-PCR)

Ipsilateral cortical and thalamic tissue from randomly selected CCI and sham-operated mice (n = 3 per group) was collected 1 week post-operation and homogenised in TRIzol (Ambion®, Thermo-Fisher Scientific). Total RNA was isolated according to the manufacturer's specifications. Complementary DNAs were synthesised using Multi Scribe Reverse Transcriptase (Applied Biosystems, Thermo-Fisher Scientific) and used for qPCR with the Power SYBR Green Master Mix (Applied Biosystems) on a 7500 RT-PCR System (Applied Biosystems; see Inline Supplemental Table for primers). Inflammatory cytokine gene mRNA levels were normalised to the levels of hypoxanthine guanine phosphoribosyl transferase.

### 2.9 Statistical analyses

Quantitative data were expressed as the mean ± standard error of the mean. Differences between experimental groups were analysed using *t*-test. All statistical analyses were performed using GraphPad Prism version 6.0 (GraphPad Software, San Diego, CA, USA). Statistical significance was defined as *p* < .05.

## 3 Results

**Fig. 1:**
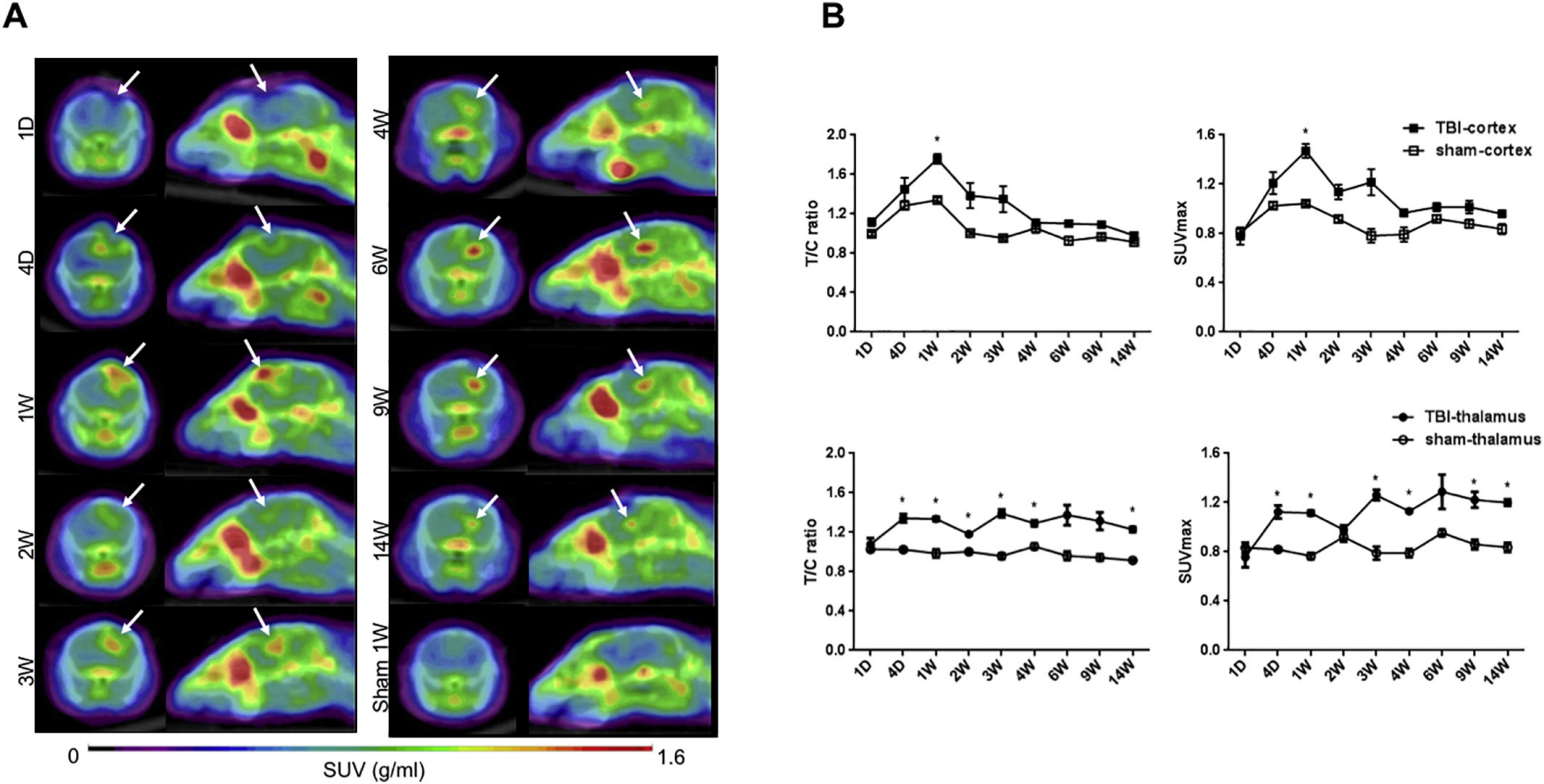
Inflammatory migration to the ipsilateral thalamus after focal cortical injury.(A)Serial ^18^F-DPA-714 PET/CT images 20 min after administration (axial: left; sagittal: right). Translocator protein uptake was initially detected in the cortex and later in the thalamus (arrow). SUV, standardized uptake value; D, day; W, week. (B)Semi-quantitative analysis of target-to-cerebellum ratio (T/C; left) and maximum standardized uptake value (SUVmax; right) for the translocator protein at the cortical injury site and the thalamus. TBI, traumatic brain injury; D, day; W, week. *, *p* < .05 compared to the sham group; *t*-test with Sidak-Bonferroni method

^18^F-DPA714 PET showed increased TSPO uptake in the focal injury site (cortex) and expansion to remote areas (ipsilateral thalamus), 1 and 3 weeks, respectively, post-TBI (Fig. 1A). Semi-quantitative analysis showed two peaks of ^18^F-DPA714 uptake in the whole brain at 7 and 21 days after injury; the first concerned the cortical and the second the thalamic uptake (target-to-cerebellum ratio: 1.76 ± 0.10 and 1.39 ± 0.07, respectively; Fig. 1B and Supplemental Fig. 1). The cortical uptakes rapidly decreased after 1 week, and the thalamus showed long sustained uptakes after 3 weeks. There was no anatomical site other than the ipsilateral thalamus showing similar accumulation. In vivo MRI only revealed a regression of the injured cortex and a slight atrophy in the ipsilateral thalamus in the time course post-TBI (Supplemental Fig. 2).

**Fig. 2:**
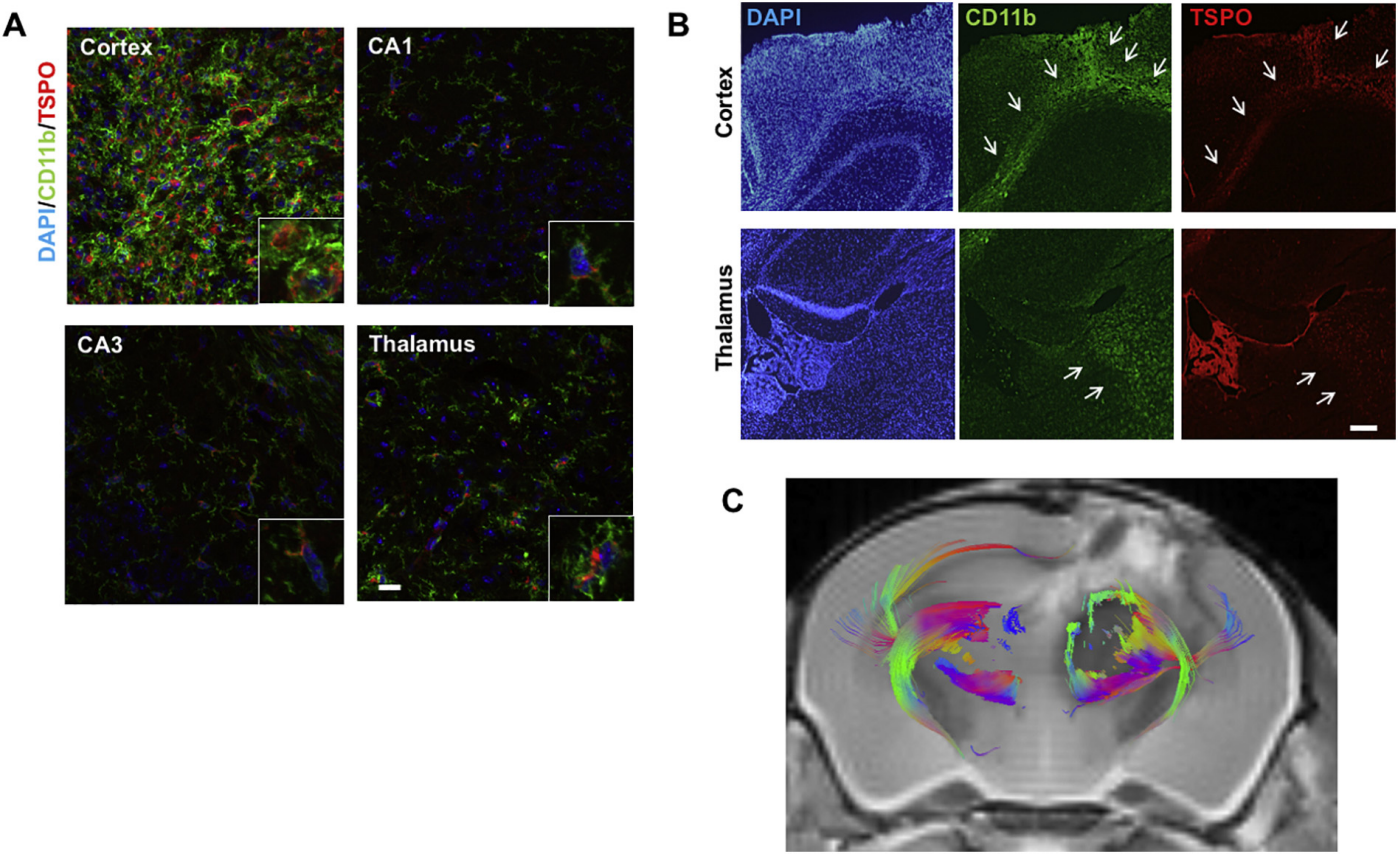
Translocator protein (TSPO) upregulation in microlia/macrophages located along the damaged white matter tracts. (A) Representative merged fluorescence images of brain sections of the ipsilateral hemisphere at 1 week post-injury stained with CD11b (green) and TSPO (red) and counterstained with DAPI (blue). Co-localization patterns of CD11b and TSPO at week 1 post-injury revealed that amoeboid cells were located almost exclusively in the ipsilateral cortex (upper left), whereas ramified cells were seen in the hippocampus (CA1: upper right; CA3: lower left). Transitional cells were observed in the ipsilateral thalamus (lower right). Representative high-magnification images are in the lower right-hand corner (squares). Note that TSPO immunoreactivity closely correlated with microglial morphological changes. Scale bar: 150 μm. (B) Representative images of coronal (axial) brain sections on caudal slices of the cavitation in the ipsilateral cortex and thalamus. Note that CD11b^+^ and TSPO^+^ cells have spread from the cortex to the thalamus via white matter tracts (arrow). Scale bar: 100 μm. (C) Ex vivo tractography at week 1 post-injury targeting the bilateral thalami. Red: medial-lateral; blue: rostral-caudal; green: dorsal-ventral.

One week post-TBI, we performed immunohistological analysis for the histological examination of TSPO cellular expression in the injured cortex. We found that TSPO mainly co-localised with amoeboid CD11b-positive microglia/macrophages and weakly with GFAP-positive astrocytes (Fig. 2A and Supplemental Fig. 3). Thalamic CD11b-positive cells exhibited more transitional morphologies and low TSPO immunoreactivity. In the hippocampal region between the ipsilateral cortex and thalamus, microglia appeared ramified with minimal TSPO expression. These CD11b-positive microglia/macrophages seemed to spread from the cortex to the thalamus via white matter tracts, rather than via the hippocampus (Fig. 2B). On the MR tractography, 1 week post-TBI, we found that corticothalamic fibre tracts were irregularly disrupted on the ipsilateral side, compared to the contralateral side (Fig. 2C), suggesting that these TSPO/CD11b positive cells accumulated in the damaged corticothalamic projections.

**Fig. 3:**
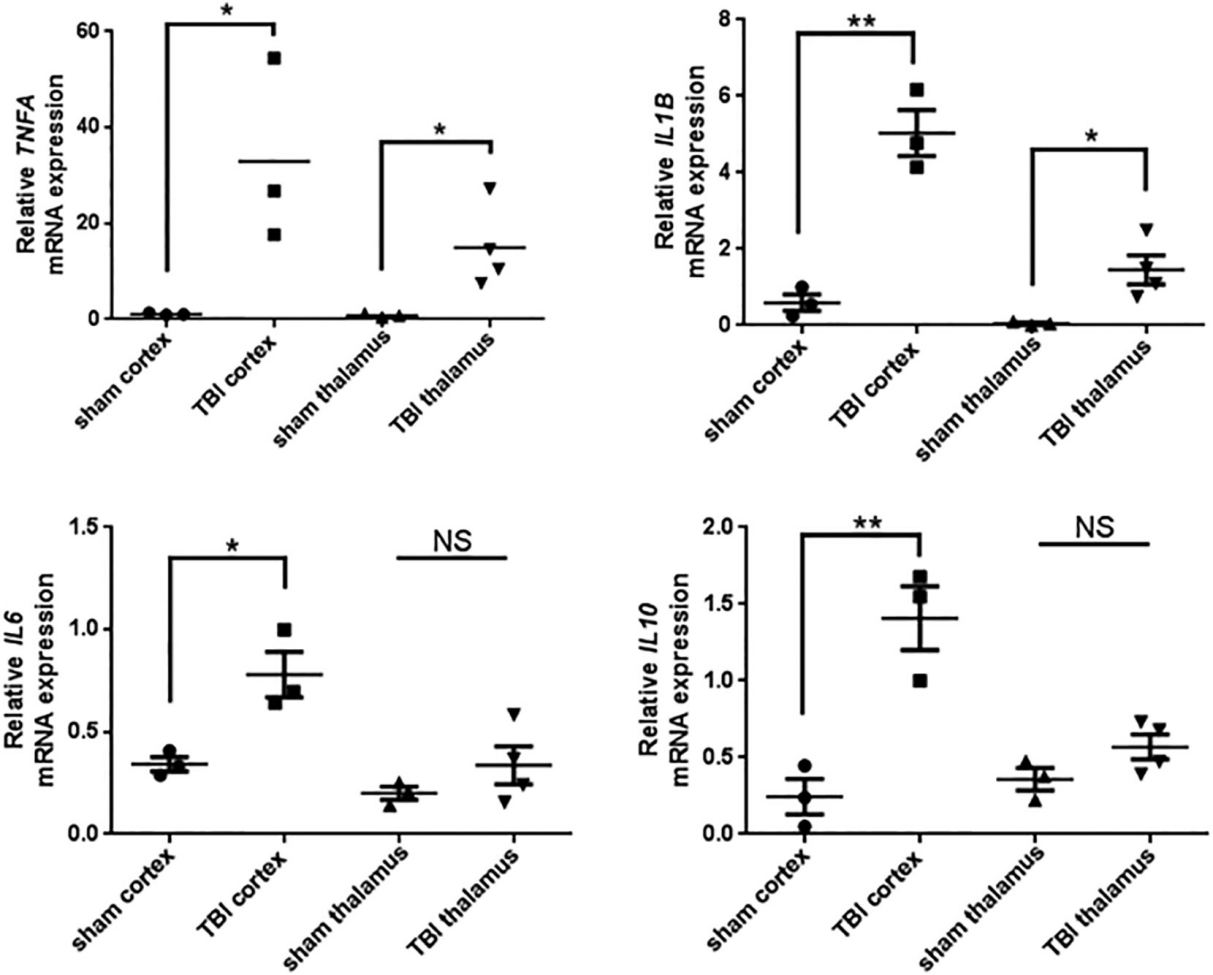
Delayed inflammation in the ipsilateral thalamus. Cytokine expression in the ipsilateral cortex and thalamus parenchyma at week 1 post-injury in sham-operated animals and animals with traumatic brain injury (TBI). TNF-α, tumor necrosis factor a; IL, interleukin. *n* = 3–4; *, *p* < .05; **, *p* < .01 compared to the sham group.

Furthermore, RT-PCR analysis at this time-point showed advanced inflammation with increased levels of all examined cytokines in the cortex. In contrast, the thalamus exhibited early inflammatory signs with slight increases in interleukin (IL)-1 beta and tumor necrosis factor-alpha (TNF-α), suggesting delayed inflammation in the ipsilateral thalamus (Fig. 3).

TUNEL fluorescent images, 1 day post-injury, showed no apoptotic cells in the thalamus, contrary to the injured cortex, suggesting no direct injury to the thalamus by the CCI (see Inline Supplemental Fig. 4). DAPI fluorescent images at 1 week also revealed that the initially damaged cortex and related white matter were necrotic, whereas the thalamic cells were still intact (Supplemental Fig. 5).

**Fig. 4:**
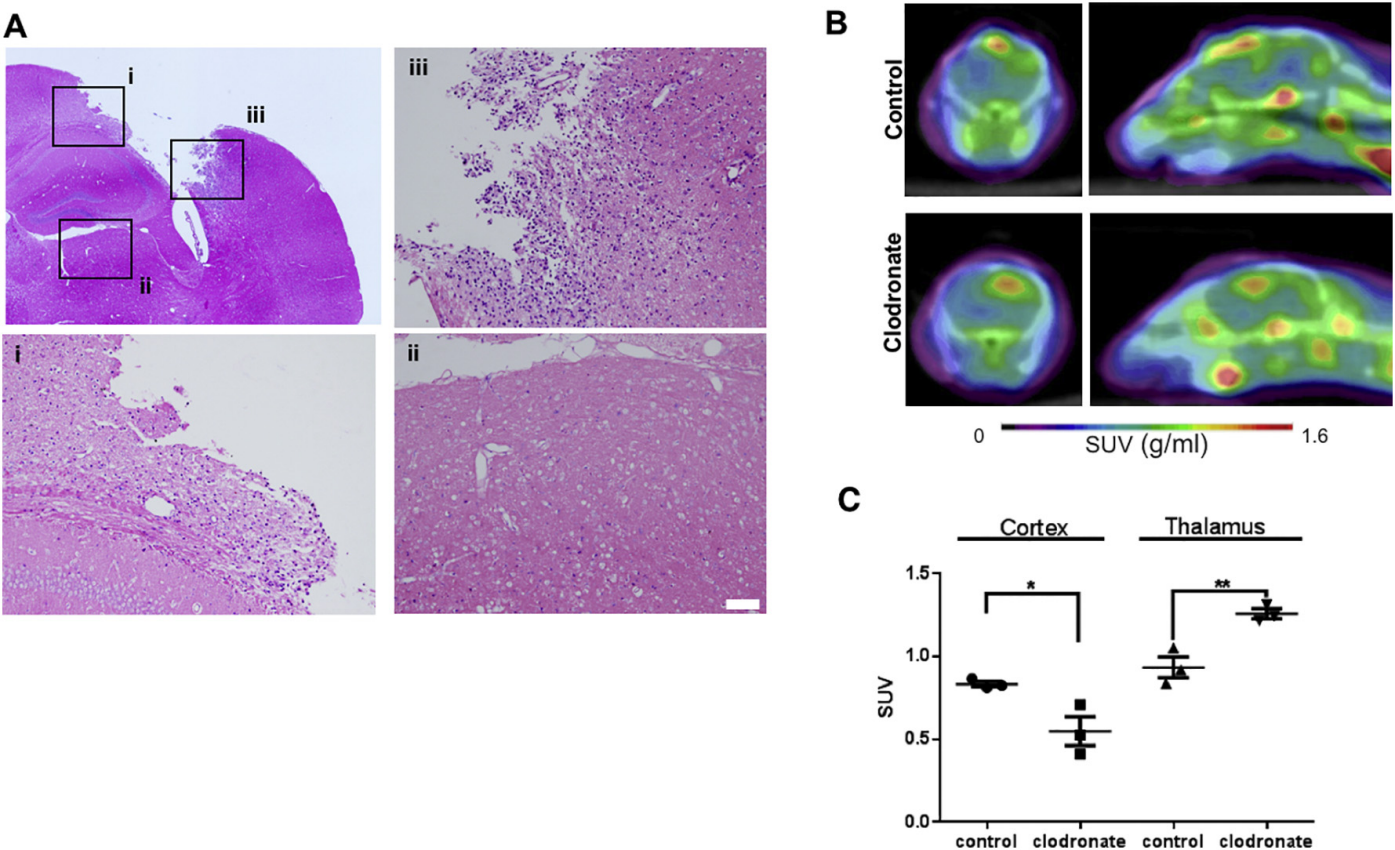
Thalamic translocator protein (TSPO) uptake induced by microglial activation. (A) Giemsa-stained axial brain slices are shown on the upper left at week 1 post-injury. (i-iii) Higher magnification micrographs of the medial cavitation (i), thalamus (ii), and lateral cavitation (iii) in the ipsilateral hemisphere. (B,C) ^18^F-DPA-714 PET/CT images at week 1 post-injury in animals subjected to controlled cortical impact (CCI), treated with control liposomes (upper panel) or clodronate liposomes (lower panel). n = 3 per group; *, *p* < .05; **, *p* < .01 compared to the control group. SUV, standardized uptake value.

Giemsa staining revealed mononuclear cells, such as phagocytic macrophages, along the cavitation boundary, although these cells were not detected in the ipsilateral thalamus (Fig. 4A). In the macrophage depletion study at week 1 post-injury, clodronate successfully suppressed TSPO uptake in the cortex, but increased it in the thalamus (Fig. 4B,C), suggesting that the cortical uptake mostly consisted of phagocytic macrophages recruited from the bloodstream, while the thalamic was most likely induced by resident microglia.

**Fig. 5:**
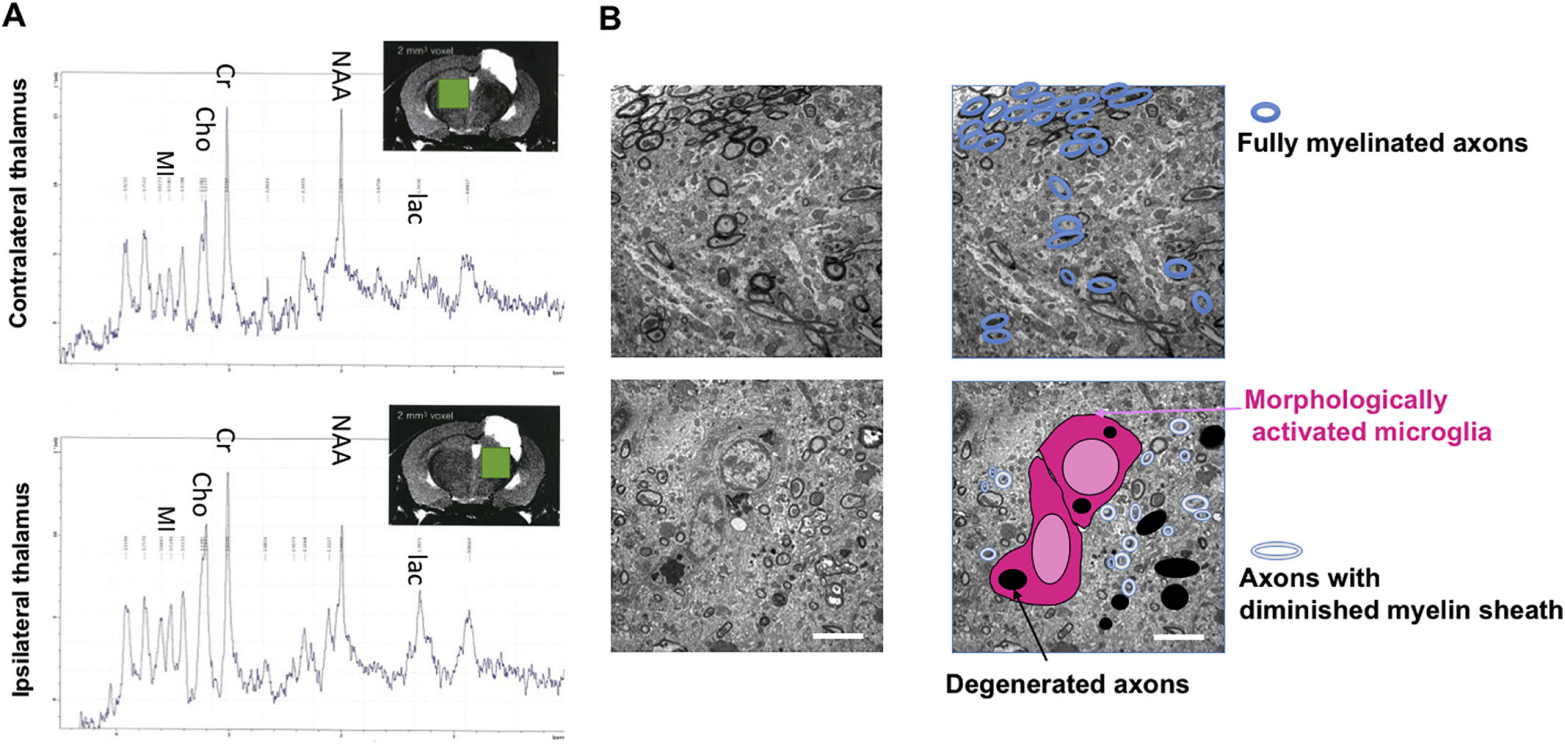
Microglial activation induces neurodegeneration in the ipsilateral thalamus in the chronic phase of inflammation. (A) Representative proton magnetic resonance (^1^H-MR) spectra at week 4 post-injury. Single-voxel MR spectrometry enabled us to selectively examine the thalamus without the influence of the surrounding tissue. MI, myo-inositol; Cho, choline compounds; Cr, creatine; NAA, *N*-acetylaspartate; Lac, lactate (B) Representative electron microscopy images at week 4 post-injury. Scale bar: 5 μm.

Finally, we performed thalamic analyses in the chronic phase. MRS at 4 weeks after injury showed that, compared to the contralateral thalamus, the ipsilateral thalamus exhibited reduced *N*-acetylaspartate and increased lactate, choline, and myo-inositol, indicating both neurodegeneration and neuroinflammation (Fig. 5A). These MRS findings persisted up to week 14 post-injury (Supplemental Fig. 6), which corresponded to the PET findings. Electron microscopy at 4 weeks post-TBI revealed morphologically activated microglia surrounding degenerated axons and poorly myelinated neurons in the ipsilateral thalamus, indicating the activation of resident microglia and damage to the remaining neurons (Fig. 5B). During the chronic phase, diffuse glial activation was observed in the ipsilateral thalamus (Supplemental Fig. 7). TSPO expression was mainly evident in active microglia in the thalamus (see Inline Supplemental Fig. 8).

## 4 Discussion

Here, we used TSPO-PET to examine acute and chronic whole-brain neuroinflammation in experimental mice with TBI, thereby emphasizing the differential localization of both the acute and chronic neuroinflammation. TSPO-PET elucidated two distinct types of inflammation occurring after focal cortical injury: acute inflammation in the vicinity of the cortical lesion, with rapid neuronal loss along the injured tracts; and delayed inflammatory response in the ipsilateral thalamus with long-term neuronal degeneration of the uninjured tracts (see Graphical Abstract).

TSPO PET showed that the cortical uptake rapidly decreased after 1 week, while the thalamus showed long sustained uptake after 3 weeks. Therefore, we considered that 1 week post-TBI was the most relevant timepoint for understanding the pathophysiology of inflammatory progression. For this reason, we performed a detailed analysis, including various histological analyses, tractography, PCR, and macrophage depletion test, at this timepoint. Cortical uptake returned to baseline levels after 4 weeks. Therefore, we considered the 4-week timepoint post-injury as the start of the chronic phase of inflammation and performed serial MRS for thalamic assessment.

To our knowledge, this study is the first to quantify the progression of inflammation to remote sites after TBI using in vivo imaging (Eierud et al., 2014). Wang and his colleagues (2014) reported increased lesion-to-normal brain ratios of ^18^F-DPA-714 in CCI rats, peaking at around 1 week and decreasing gradually to nearly normal levels by 4 weeks. In contrast, our study showed continued inflammation in the thalamus, although this deep structure of the brain was not initially involved in the injury. The major difference between the two studies is that we induced injury over the left sensorimotor cortex, the origin of cortico-thalamic projections. More specifically, our cortical injury included layer 6 of the neocortex, which contains cells that project to the sensory or thalamic nuclei (West et al., 2006). Thus, depending on the location and severity of the injury, inflammation might not progress to the subcortex. Johnson and his colleagues revealed clusters of activated microglia in 28% of patients with single brain injury who survived for >1 year (Johnson et al., 2013). Our study might shed light on the mystery as to why not all TBIs result in persistent inflammation.

Previous studies on TBI have mainly used the first-generation TSPO-PET tracer, ^11^C-PK-11195, to evaluate glial activation in small animals, as well as in humans. Nevertheless, ^11^C-PK-11195 use is limited due to its high non-specific binding (Kreisl et al., 2010). Second generation TSPO-PET tracers, such as ^11^C-DPA-713 and ^18^F-DPA-714, have the advantage of higher specific binding (Doorduin et al., 2009; Kobayashi et al., 2017). However, the inflammatory cells responsible for TSPO uptake in each progression phase after TBI have not been clarified, as TSPO expression is observed both among the activated microglia/macrophages and in reactive astrocytes (Lavisse et al., 2012). Our immunohistochemistry results showed that CD11b-positive cells are the main cellular correlate to TSPO uptake beyond reactive astrocytes, consistent with results of previous studies, using ^18^F-DPA-714 ligand in a weight-drop closed head injury mouse model (Israel et al., 2016), fluid percussion injury model in rats (Cao et al., 2012), and focal cerebral ischemia injury rat model (Martín et al., 2010). However, the lack of a specific antibody to differentiate microglia from macrophages prevented us from distinguishing local microglia from circulating macrophages, recruited to the brain post-injury. We used two methods to identify peripheral monocyte-macrophages or resident microglia, as the main cells correlating to TSPO uptake: Giemsa staining, normally used as a leukocyte stain; and intraperitoneal administration of clodronate liposomes, used to ablate peripheral macrophages and monocytes (Rooijen and Sanders, 1994). Because clodronate liposomes cannot cross the blood-brain barrier, they cannot ablate phagocytic microglia in the brain, unless they are delivered directly in the brain (Fulci et al., 2007). In this way, we proved that TSPO-PET with ^18^F-DPA-714 reflects the acute cortical inflammation, induced by phagocytic macrophages, as well as the thalamic inflammation from local microglia at 1 week post injury. We confirmed the sensitivity of TSPO-PET, as our immunohistochemistry showed that TSPO uptake well reflects the different morphological states of microglia (ramified, transitional, and amoeboid). As a semi-quantitative approach, we used the T/C ratio, based on a previous human study (Arlicot et al., 2012). In a previous preclinical study, the cerebellum was evaluated as a reference region, which improved the variability compared to the average SUV analysis (Takkinen et al., 2017; Rominger et al., 2013). In our study, the TSPO uptake rates in the cerebellum were comparable between the TBI and control mice (SUVmean = 0.87 ± 0.09 and 0.86 ± 0.09, respectively). This suggests that no significant inflammatory effect existed in the cerebellum, and that it could be used as a reference region. However, we should be careful in the evaluation involving the cerebellum as a reference region, as TSPO expression could be present throughout the brain.

One week after injury, we could only detect a single uptake peak at the initial injury site. In agreement, it was reported that glial scar formation around the central lesion core, not involving prolonged tissue damage, is basically completed by 2–4 weeks after the acute insult (Burda and Sofroniew, 2014). However, although the thalamus was not initially involved in the injury, we observed mild increases in TSPO uptake between day 4 and 7 post-injury. Immunostaining results showed that this increase reflects microglia with transitional morphologies, such as changing to amoeboid form, upon activation in the thalamus. RT-PCR results at week 1 post-injury corroborated this finding, as CCI significantly increased gene expression of all cytokines examined in the cortex (progressive inflammatory phase), while the ipsilateral thalamus exhibited increased gene expression for the primary response cytokines IL-1β and TNF-α, without significant changes in the expression of the secondary response cytokine IL-6 or the anti-inflammatory cytokine IL-10 (early inflammatory phase). Tractography showed the anatomical connection between these two sites, the initial injury site in the cortex and the ipsilateral thalamus, being the cortico-thalamic projection. Previous findings indicate that pathophysiological conditions characterized by synaptic input loss, secondary to acute axon destruction, may result in localised inflammation around the downstream target neuron (Banati et al., 2000). We conclude that thalamic microglial activation in the acute phase might be initiated by axon loss or cell death of damaged cortico-thalamic projections.

In the chronic phase, cortical TSPO uptake almost returned to baseline; however microglia continued to be activated in the ipsilateral thalamus. Activated microglia can polarise into M1 (pro-inflammatory) or M2 (anti- inflammatory) phenotypic states, depending on the nature of the inflammatory stimulus (Tang and Le, 2016). M1 microglia produce and release cytokines that can aggravate neural injury. In contrast, M2 microglia release neurotrophic factors but also exert phagocytic roles, thus enabling neuronal repair (Tang and Le, 2016). Accordingly, it remains unclear whether prolonged moderate TSPO uptake, indicating microglial activation, is neuroprotective or neurodestructive. We addressed these questions, by using serial ^1^H-MRS to directly assess the neurochemical profiles in the bilateral thalami. A study on TBI found decreased levels of *N*-acetylaspartate in the injured cortex, whereas the perilesional hippocampus appeared normal, suggesting defective mitochondria or neurodegeneration (Harris et al., 2012). The authors also found high levels of lactate, indicative of hypoxia, and of choline and myo-inositol, indicative of membrane damage or inflammation (Harris et al., 2012). Our MRS results, confirmed by electron microscopy findings, elucidated that chronic microglial inflammation has destructive effects on the remaining neurons. The thalamus has direct, dense, and reciprocal anatomical connections with all parts of the cerebral cortex (Cho et al., 2015). Histological analyses and ex vivo MRI showed that chronic inflammation and degeneration include the ipsilateral thalamus, and particularly the laterodorsal and lateral posterior thalamic nuclei, which provide inputs and outputs to other cortical areas. We suggest that the sustained activation of thalamic microglia, triggered by the damage of the cortico-thalamic projections, results in the continued neurodegeneration of related neuronal networks. In line with our findings, Gregory and colleagues used ^11^C-PK 11195 and diffusion MRI and demonstrated that the degree of thalamic ^11^C-PK 11195 binding and thalamic fractional anisotropy in patients with TBI are closely related with damages in the thalamo-cortical tract (Scott et al., 2015). Moreover, clinical evidence has shown that cognitive dysfunction in patients with TBI correlates with thalamic damage (Little et al., 2010; Ramlackhansingh et al., 2011), regardless of the injury site. This would justify future animal and clinical studies using PET imaging to examine possible treatment of thalamic inflammation before it progresses to post-TBI chronic neurodegeneration.

The present study has certain limitations. First, we did not analyse the BBB breakdown in the ipsilateral thalamus. Following TBI, pro-inflammatory cytokines, like IL-1β, IL-6, and TNFα contribute to increased BBB permeability (Chodobski et al., 2011). Breakdown or increased permeability in the BBB would increase the access of the PET tracer to the brain parenchyma. We also did not analyse the cerebral blood flow after TBI. The distribution of PET tracers is influenced by blood flow after TBI (Cselényi and Farde, 2015), and changes in cerebral blood flow would result in atrophy. Eliminating these impacts on the results via using kinetic modelling or separate measurements with MRI would be useful in future studies. Second, the sample size was small. However, to prove our hypothesis we used a multimodal approach, employing different methods, to gain a broader, more comprehensive view. Furthermore, the extent of injury was consistent in this model of TBI (Ueno et al., 2012), and our data had low variability, indicating that larger samples would not likely improve the quality of the results. Third, we did not completely rule out the influence of the initial injury on the ipsilateral thalamus. There might be other mechanisms, such as concussion, than those discussed above. Lastly, we did not determine the mechanism by which clodronate liposomes exacerbated the inflammation in the thalamus. Nevertheless, this strongly suggests that anti-inflammatory treatment solely restricted to areas proximal to the TBI site may be ineffective. Future studies are required to explore therapeutic interventions, including anti-inflammatory agents and their relation to microglial phenotypes.

Treating TBI related progressive disorders requires a comprehensive understanding of inflammatory initiation, evolution, resolution, and the respective impact on neural networks. The present findings provide strong evidence that injury-induced inflammation expands to remote sites via neuronal projections. Therefore, we propose the term “brain injury-related inflammatory projection” (BIRIP) to describe the tract-related chronic neuroinflammatory pathology. TSPO-PET allows us to determine the location and degree of microglial activation and identify non-resolving neuroinflammation, as a potentially important mediator of post-TBI neurodegeneration.

## 5 Conclusion

We found that thalamic neuroinflammation persists even after the inflammatory response diminishes in the primary injury site in the cortex, leading to further brain network disorganization and dysfunction. Using a multimodal approach, our study provided a more detailed characterization and improved interpretation of TSPO signals after focal TBI. TSPO PET imaging is a useful tool to investigate prolonged inflammatory reactions and provides the potential of monitoring pharmacological anti-inflammatory treatment.

## Appendix A Supplementary data

Supplementary material Image 1
