# Supplementary material for: Inflammatory projections after focal brain injury trigger neuronal network disruption: An 18F-DPA714 PET study in mice
Source: Neuroimage Clin. 2018 Oct 1;20:946–54. doi: 10.1016/j.nicl.2018.09.031 (PMC6178196; doi:10.1016/j.nicl.2018.09.031)
Supplement: Supplementary file 1 — Supplementary material [file mmc1.docx]

Inflammatory projections after focal brain injury trigger neuronal network disruption: an ^18^F-DPA714 PET study in mice

Sanae Hosomi*, Tadashi Watabe, Yuki Mori, Yoshihisa Koyama, Soichiro Adachi, Namiko Hoshi, Mitsuo Ohnishi, Hiroshi Ogura, Yoshichika Yoshioka, Jun Hatazawa, Toshihide Yamashita, Takeshi Shimazu

***Corresponding author**

Sanae Hosomi, MD

2-15 Yamada-oka, Suita-shi, Osaka 565-0871, Japan

Tel: +81-6-6879-5707, Fax: +81-6-6879-5720

E-mail: s-hosomi@hp-emerg.med.osaka-u.ac.jp

**Electronic Supplementary Material**

**Contents:**

- 3 Supplemental methods
- 1 Supplemental Table
- 8 Supplemental Figures
- 1 Supplemental reference

**Supplemental Materials and Methods**

***Terminal deoxynucleotidyl transferase dUTP nick nick-end labeling (TUNEL)***

Cells with fragmented DNA were detected using a TUNEL apoptosis detection kit (Chemicon® Merck Millipore, Darmstadt, Germany) according to the manufacturer’s specifications. Briefly, fragmented DNA was labelled with digoxigenin-dUTP in the presence of TdT enzyme. Rhodamine-conjugated anti-digoxigenin antibody was used to visualise apoptotic nuclei. Sections were counterstained with DAPI.

MRI and MRS

Images and spectra were acquired using the same 11.7-tesla scanner and 15-mm inner-diameter coil described in section 2.4.

***In vivo MRI & MRS***

*In vivo* T_2_-weighted MR images were acquired from the same mouse (n = 3) at different time-points post-injury using the following parameters: repetition time/echo time (TR/TE) = 5000 ms/37 ms, flip angle (FA) = 180°, number of signals averaged (NSA) = 8, field of view (FOV) = 16 mm × 16 mm, matrix size = 256 × 256, slice thickness = 0.3 mm, and acquisition time = 11 min. *In vivo* ^1^H-MRS was performed to measure the detailed neurochemical profile of TBI-operated animals (n = 3) at 4, 6, 9, and 14 weeks after brain injury, using the same animals at each time-point. Diffusion-weighted images were obtained using a spin echo sequence (SE) with the following parameters: TR/TE = 5000 ms/14 ms, NSA = 2, FOV = 16 mm × 16 mm, matrix size = 128 × 128, slice thickness = 0.3 mm, b-value = 1000 s/mm^2^, and acquisition time = 21 min.

***Ex vivo MRI***

In order to obtain detailed information that would not be possible using *in vivo* MRI, we performed *ex vivo* diffusion weighted MRI (DWI) on fixed brain tissues, as described previously (Lerch et al., 2012). In brief, mice were deeply anesthetized with medetomidine hydrochloride (0.3 mg/kg), midazolam (4 mg/kg), and butorphanol (5 mg/kg); intracardiac perfusion was then performed with Dulbecco's phosphate-buffered saline (PBS) followed by 4% paraformaldehyde (PFA) for fixation. Brains were harvested, post-fixed overnight in 4% PFA, and suspended in PBS. Diffusion weighted images were obtained using an SE sequence with the following parameters: TR/TE = 5000 ms/21 ms, NSA = 16 or 20, field of FOV = 15 × 15 mm (axial) or 20 x 20 mm (sagittal), matrix size = 512 × 512, slice thickness = 0.3 mm, b-value = 1000 s/mm^2^, and acquisition time = 11 hr 23 min or 13 hr 53 min.

**Supplemental Table.** Primers used for quantitative PCR

| Primer | Sequence (5′→3′) |
| --- | --- |
| TNF-α-forward | AAAATTCGAGTGACAAGCCTGTAG |
| TNF-α-reverse | CCCTTGAAGAGAACCTGGGAGTAG |
| IL-1β-forward | ATGATAACCTGCTGGTGTGTGA |
| IL-1β-reverse | TTTGTCGTTGCTTGGTTCTCC |
| IL-6-forward | GACAAAGCCAGAGTCCTTCAGAGAGATACAG |
| IL-6-reverse | TTGGATGGTCTTGGTCCTTAGCCAC |
| IL-10-forward | CCAAGCCTTATCGGAAATGA |
| IL-10-reverse | TCTCACCCAGGGAATTCAAA |
| HPRT-forward | GTTGGATACAGGCCAGACTTTGTTG |
| HPRT-reverse | CCAGTTTCACTAATGACACAAACG |

PCR, polymerase chain reaction; HPRT, hypoxanthine guanine phosphoribosyl transferase; TNF, tumour necrosis factor; IL, interleukin

**Supplemental Figure legends**

**
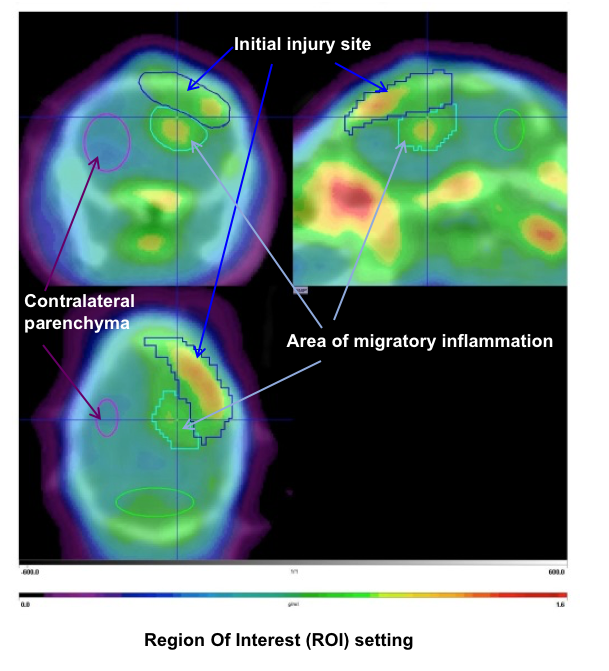
**

**Supplemental Fig. 1 Translocator protein uptake analysis using positron-emission tomography.** Areas outlined in dark blue and light blue represent the regions of interest for the initial cortical injury site and the thalamic site of inflammation, respectively. Red and green areas indicate the contralateral unaffected regions and the cerebellum, respectively


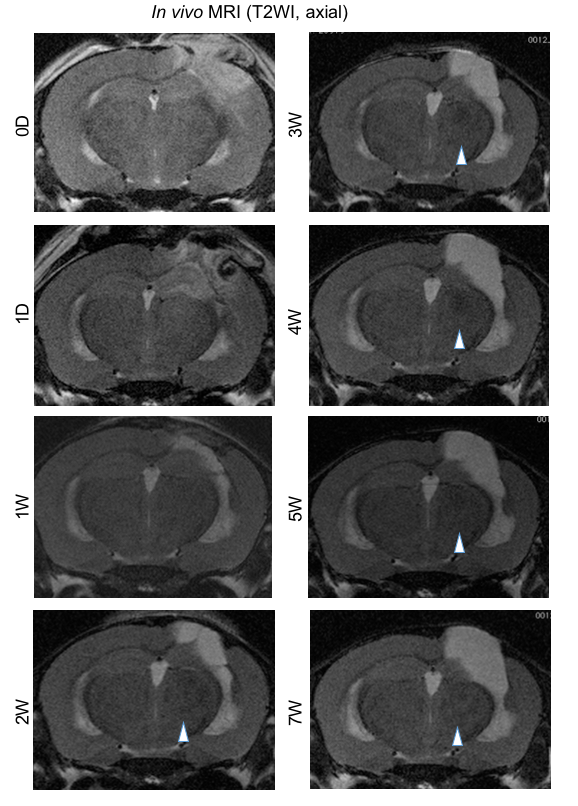


**Supplementary Fig. 2 Chronic degeneration in the ipsilateral thalamus observed in *in vivo* MRI.** *In vivo* T2-weighted magnetic resonance images. Note the slight atrophy (arrow head) and enlarged ventricle in the thalamus. D, day; W, week.

**
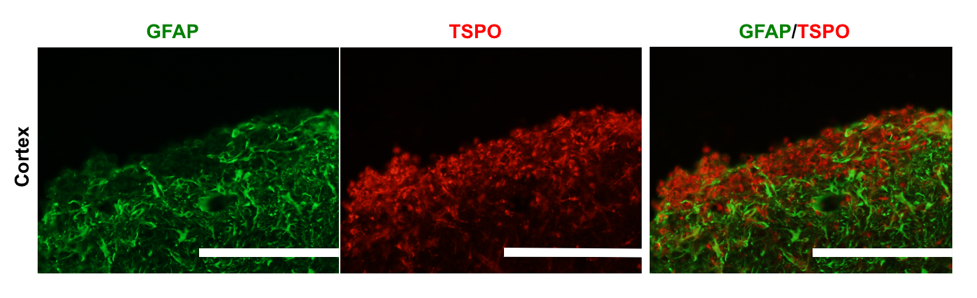
**

**Supplemental Fig. 3 Analysis of localization of translocator protein (TSPO) and astrocyte at the initial cortical injury.** Representative fluorescence images of a brain section showing glial fibrillary acidic protein (GFAP)-labelled cells (green) and TSPO-positive cells (red) in the ipsilateral cortex at 1 week post-injury. Scale bar: 100 μm


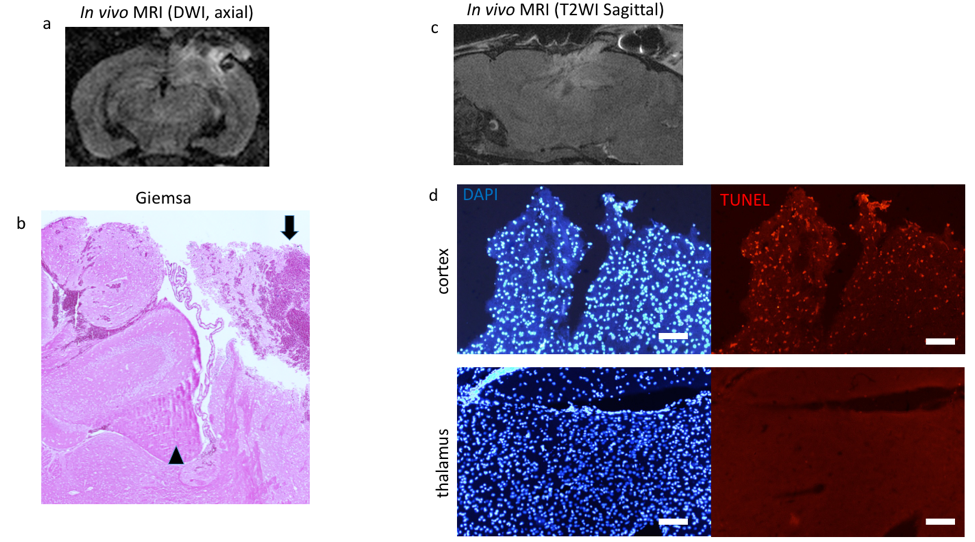
**Supplemental Fig. 4 Analysis of initial cortical injury.** (A) Representative diffusion-weighted images using magnetic resonance imaging (MRI), showing cellular swelling with high intensity in the injured cortex and adjacent hippocampus, which did not occur in the ipsilateral thalamus. (B) Coronal (axial) Giemsa staining showing cortical hematomas (arrow) and bleeding along the corpus callosum. On the ipsilateral side, the fimbria of the hippocampus was distended (arrowhead), but no microbleeding was observed in the thalamus. (C) A representative T2-weighted sagittal image also shows no signs of thalamic injury. (D) Representative sagittal images of terminal deoxynucleotidyl transferase dUTP nick-end labelling (TUNEL) in the injured cortex and ipsilateral thalamus. An overabundance of TUNEL-positive apoptotic cells was present in the injured cortex. In contrast, no TUNEL-positive cells were observed in the ipsilateral thalamus of TBI mice. Scale bar: 50 μm


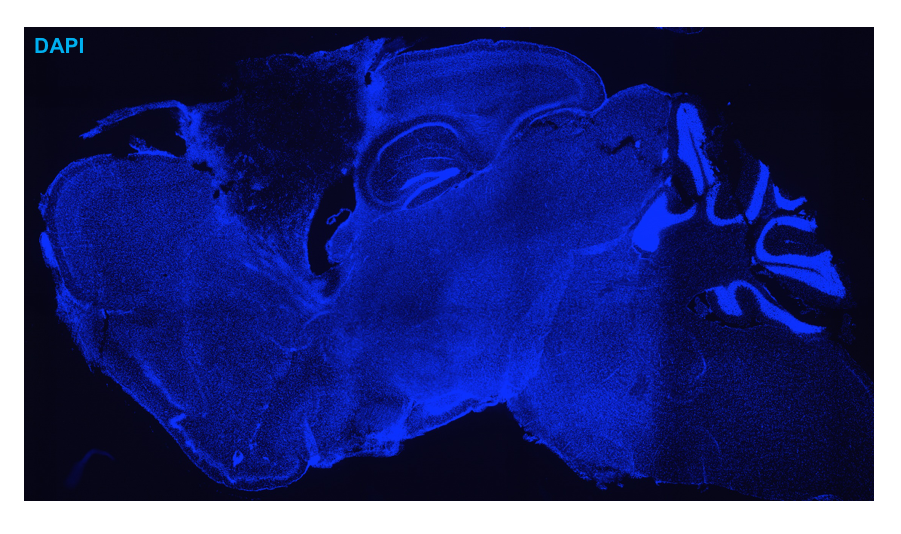
**Supplemental Fig. 5** **Necrosis in the injured cortex.** Representative images of sagittal brain sections stained with DAPI (blue) at 1 week post injury showing the injury site.


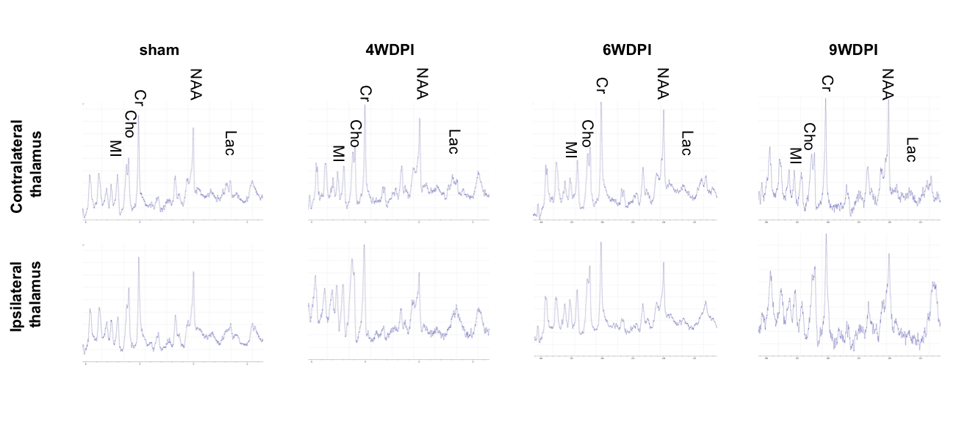


**Supplemental Fig. 6** Chronic neuroinflammation and neurodegeneration in the ipsilateral thalamus. Serial proton magnetic resonance (^1^H-MR) spectra from week 4 post-injury. Compared to the contralateral thalamus, the ipsilateral thalamus exhibits reduced N-acetylaspartate (NAA) and increased lactate (Lac), choline compounds (Cho), and myo-inositol (MI). Cr, creatine; WPI, weeks post injury.

**
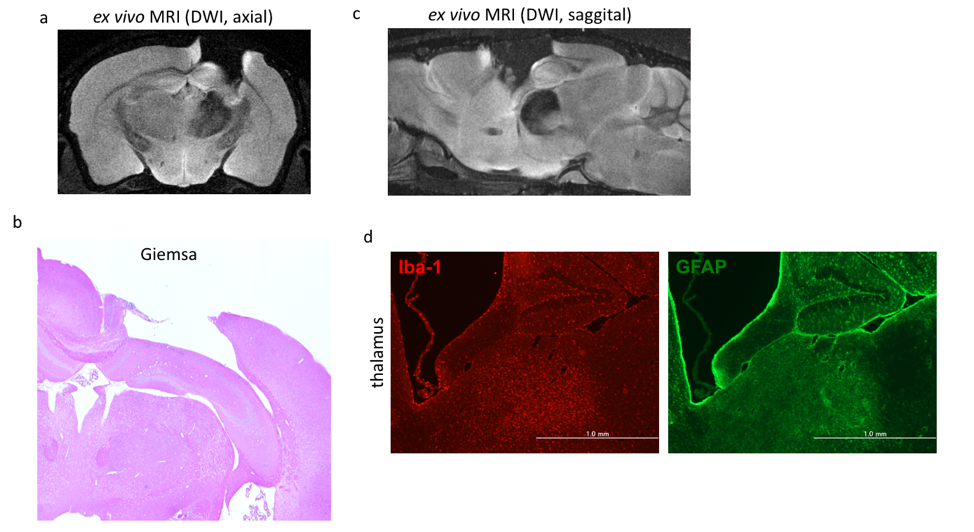
**

**Supplemental Fig. 7 Diffuse glial activation and on-going neurodegeneration in the ipsilateral thalamus.** Representative images of sagittal mouse brain sections showing ionized calcium-binding adaptor molecule 1-positive microglia (red) and glial fibrillary acidic protein-positive astrocytes (green) in the ipsilateral cortex and thalamus at 6 weeks post-injury (A). Scale bar: 1 mm. (B) *Ex vivo* diffusion-weighted images revealing distinct damage patterns in the injured cortex and ipsilateral thalamus. By 6 weeks post-injury, the cortex shows a cavity surrounded by hyperintense scarring. In contrast, the ipsilateral thalamus is hypointense, suggesting a chronically progressive degenerative process.

**
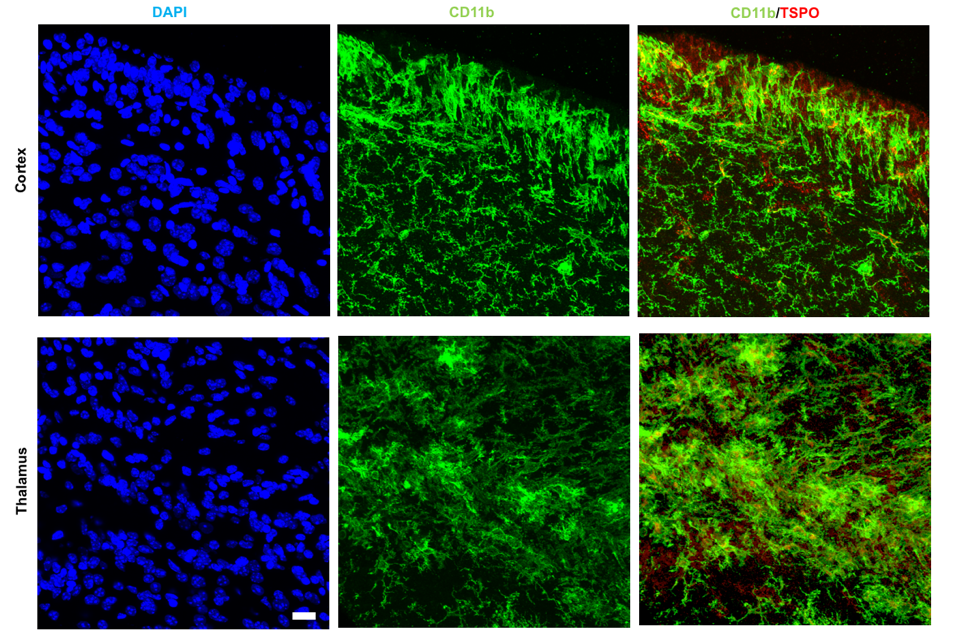
Supplemental Fig. 8 Localization patterns of CD11b and translocator protein (TSPO) in the chronic phase.** Representative fluorescence images of brain sections at the level of the ipsilateral cortex and thalamus at 6 weeks post-injury stained with CD11b (green) and TSPO (red) and counterstained with DAPI. TSPO immunoreactivity co-localizes with CD11b, marking activated microglia in the ipsilateral thalamus, and is evident in the glial scaring of the ipsilateral cortex. Scale bar: 50 μm

**Supplemental Reference**

Lerch, J.P., Gazdzinski, J., Germann, J., Sled, J.G., Henkelman, R.M., and Nieman, B.J., 2012. Wanted dead or alive? The tradeoff between in-vivo versus ex-vivo MR brain imaging in the mouse. Front. Neuroinform. 6, 6.
